# Dual effects of obesity on satellite cells and muscle regeneration

**DOI:** 10.14814/phy2.14511

**Published:** 2020-08-09

**Authors:** Ashley E. Geiger, Morgan R. Daughtry, Con‐Ning Yen, Laila T. Kirkpatrick, Hao Shi, David E. Gerrard

**Affiliations:** ^1^ Department of Animal and Poultry Sciences Virginia Polytechnic Institute and State University Blacksburg VA USA

**Keywords:** muscle regeneration, obesity, satellite cell

## Abstract

Obesity is a complex metabolic disorder that often leads to a decrease in insulin sensitivity, chronic inflammation, and overall decline in human health and well‐being. In mouse skeletal muscle, obesity has been shown to impair muscle regeneration after injury; however, the mechanism underlying these changes has yet to be determined. To test whether there is a negative impact of obesity on satellite cell (SC) decisions and behaviors, we fed C57BL/6 mice normal chow (NC, control) or a high‐fat diet (HFD) for 10 weeks and performed SC proliferation and differentiation assays in vitro. SCs from HFD mice formed colonies with smaller size (*p < *.001) compared to those from NC mice, and this decreased proliferation was confirmed (*p < *.05) by BrdU incorporation. Moreover, in vitro assays showed that HFD SCs exhibited diminished (*p < *.001) fusion capacity compared to NC SCs. In single fiber explants, a higher ratio of SCs experienced apoptotic events (*p < *.001) in HFD mice compared to that of NC‐fed mice. In vivo lineage tracing using H2B‐GFP mice showed that SCs from HFD treatment also cycled faster (*p < *.001) than their NC counterparts. In spite of all these autonomous cellular effects, obesity as triggered by high‐fat feeding did not significantly impair muscle regeneration in vivo, as reflected by the comparable cross‐sectional area (*p* > .05) of the regenerating fibers in HFD and NC muscles, suggesting that other factors may mitigate the negative impact of obesity on SCs properties.

## Graphical abstract

In non‐injured muscle, muscle satellite (stem) cells from mice fed a high fat diet (HFD) exhibited reduced capacity in proliferation, differentiation, and quiescence maintenance, with a concomitant increase in apoptosis. When muscle was injured, HFD muscle recovered to an extent comparable to the lean muscle, suggesting in vivo mechanisms may exist, perhaps at the whole body level, that can mitigate or overcome damage to muscle satellite cells.

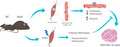

## 1 INTRODUCTION

Obesity is a metabolic disease characterized by an accumulation of adipose tissue in the body due to a positive energy balance in which energy intake is greater than energy expenditure (Serra, Mera, Malandrino, Mir, & Herrero, 2013). Prevalence of obesity in the population has dramatically increased over the past several decades worldwide and has been linked to the occurrence of cardiovascular disease, Type 2 diabetes mellitus, renal dysfunction, asthma, sleep disorders, infertility, and others (Jung, 1997; Pi‐Sunyer, 1991). Along with these pathologies, obesity is linked to an increased risk of metabolic syndrome, which is prominently defined in humans as the development of insulin resistance (Johnson, Milner, & Makowski, 2012). Physiologically, insulin resistance is characterized as decreased insulin‐stimulated blood glucose uptake by skeletal muscle and adipose tissue, as well as a failure to inhibit lipolysis and glucose production in the liver (Johnson et al., 2012; Jung & Choi, 2014). Because skeletal muscle is the most abundant insulin‐sensitive tissue and handles 75%–95% of all mediated glucose utilization (Stump, Henriksen, Wei, & Sowers, 2006), it is not surprising that a dysregulation in body metabolism affects muscle metabolism as well. Excess lipid accumulation in adipose tissues and ectopic lipid accumulation in skeletal muscle affect not only muscle insulin signaling, but also muscle maintenance and regeneration (Akhmedov & Berdeaux, 2013). Although the underlying mechanisms causing an impairment in muscle repair are not fully understood, it has been suggested that muscle satellite cells could be impacted negatively by obesity.

Satellite cells (SCs), also known as muscle stem cells, are myogenic precursors that reside on the outside of the muscle fiber between the sarcolemma and basal lamina (Hawke & Garry, 2001; Mauro, 1961; Seale & Rudnicki, 2000). SCs are responsible for the maintenance, growth, and repair of adult skeletal muscle, and the loss or impairment of SCs has been attributed to impaired muscle regeneration after damage (Montarras et al., 2005; Rudnicki, Le Grand, McKinnell, & Kuang, 2008; Tedesco, Dellavalle, Diaz‐Manera, Messina, & Cossu, 2010). SCs are normally quiescent and are activated in response to external stimuli such as growth factors, hormones, or muscle injury (Yin, Price, & Rudnicki, 2013). Once activated, SCs proceed through the myogenic lineage pathway to proliferate and differentiate to form new myofibers or repair damaged muscle fibers, while a small subpopulation of SCs self‐renew into a quiescent state to maintain SC populations (Dumont, Wang, & Rudnicki, 2015). SC lineage progression is governed through a tight spatial and temporal regulation involving a cascade of transcription factors such as paired box transcription factor 7 (Pax7) and myogenic regulatory factors (MRFs) (Seale et al., 2000). Pax7 is expressed in the majority of SCs and is widely used as a marker of SC quiescence (Seale et al., 2000). In contrast, cells expressing MRFs, such as MyoD, Myf5, MRF4, and myogenin are no longer considered quiescent (Bentzinger et al., 2008). Thus, the ability of SCs to activate, proliferate, and differentiate in response to stimuli is essential in skeletal muscle repair and regeneration post injury. Likewise, SCs must retain the ability to self‐renew after an activation event and revert to a quiescence state to maintain the regenerative capacity of the tissue should conditions necessitate repair or growth during the life span of an individual.

An accumulating body of evidence has shown that the behaviors of SCs, like stem cells in other tissues, are governed by the signals emanated from their niche environment (Brack & Rando, 2012; Cheung & Rando, 2013). In an obesity scenario, increased ectopic lipids can lead to lipotoxicity, i.e., excessive deposition of long‐chain acyl CoAs, diacyglycerols, and ceramides in the skeletal muscle (Adams et al., 2004; Holland et al., 2007; Hulver et al., 2003; Kusminski, Shetty, Orci, Unger, & Scherer, 2009; Magnusson, Friberg, Sjovall, Malm, & Chen, 2008; Turinsky, Bayly, & O'Sullivan, 1990; Turner et al., 2013). Lipotoxicity and its associated metabolites have been shown to negatively impact myogenesis. For example, ceramides can impair L6 myogenesis through phospholipase D‐mediated inhibition of myogenic transcription factor myogenin expression (Mebarek et al., 2007). Other obesity‐associated factors, such as myostatin, inflammatory cytokines including TNFα and IL‐1β, free fatty acids, and leptin, are suggested to be involved in the regulation of SC proliferation and differentiation post muscle injury (Akhmedov & Berdeaux, 2013). However, most studies on the impact of obesity on SC behaviors have used in vitro model involving cell lines, thus the results need to be cautiously interpreted since the in vivo situation is far more complex than cell culture environment. To this end, we employed an established dietary obesity model to determine SC function in vitro, ex vivo, and in vivo.

## 2 MATERIALS AND METHODS

### 2.1 Overall experimental design

In this paper, we first established an HFD‐induced obesity model, then used this model to study SC properties in vitro, ex vivo, and in vivo. In vitro studies were focused on SC proliferation, fusion and differentiation, and self‐renewal. Ex vivo study included isolating single muscle fibers and stained the fibers with cleaved caspase 3 to evaluate SC apoptosis. We then used a H2B‐GFP mouse model to track SC number and quiescent status in vivo. Finally, we employed a chemical‐induced muscle regeneration model to study the functional role of SC in vivo.

### 2.2 Mouse model

H2B‐GFP mice were purchased from the Jackson Laboratory (Bar Harbor, ME). H2B‐GFP mice express human histone 1, H2BJ, protein, fused with green fluorescent protein (GFP). The expression of the fusion protein is under the control of a tetracycline‐responsive promoter, i.e., feeding the mice with food containing tetracycline analog doxycycline (pulse period) will induce the expression of H2B‐GFP. With doxycycline food withdrawal (chase period), GFP will be retained in rarely dividing cells. We used this mouse strain to monitor SC dividing/cycling or quiescent status in vivo.

To establish an HFD‐induced dietary obesity model, C57BL/6J mice were fed either a normal chow (NC) or high‐fat diet (HFD) starting at either 4 or 6 weeks of age (Wang & Liao, 2012). The HFD formula consists of 60% of energy from fat (Teklad, East Millstone, NJ) and mice were allowed ad libitum access to the diet for at least 10 wks. Mice were weighed each week until euthanasia. All animal procedures were approved by the Virginia Tech Institutional Animal Care and Use Committee.

### 2.3 Tissue sample collection

Mice were euthanized by carbon dioxide (CO_2_) followed by cervical dislocation. *Tibialis anterior* (TA) and *gastrocnemius* (GA) muscles were collected, placed in freezing compound (O.C.T. Thermo Fisher Scientific, Fisher Healthcare, Houston, TX) and immediately frozen in isopentane precooled in liquid nitrogen. Samples were stored in −80°C until analyses. Ten micron thick sections were cut using a Microm HM550 cyrostat (Thermo Fisher Scientific, Waltham, MA) and mounted on 3‐aminoproprytriethoxysilanesy‐coated (silane, Sigma‐Aldrich, St. Louis, MO) slides for immunocytochemistry or histology.

### 2.4 Satellite cell isolation and culture

Immediately after euthanasia, mice were rinsed briefly in 70% ethanol and muscles from the hind limbs, lower back, and diaphragm were exposed, incised, and transferred to sterile phosphate‐buffered saline (PBS). Muscles were washed, and excess connective tissue, adipose tissue, blood, and hair were removed. Pooled muscles were then dissected and minced with sterile scissors to yield a fragmented muscle suspension. Muscle suspensions were digested in Ham's F10 medium (Fisher Scientific, Hampton, NH) containing 10% horse serum (Invitrogen, Carlsbad, CA) and collagenase II (500 units per mL; Invitrogen) in a 15 ml centrifuge tube for 90 min at 37°C under agitation. After a 90 min digestion, digests were triturated 20 times to separate the single fibers using a 10 ml serological pipette. Digestions were then centrifuged at 500 X g for 1 min to pellet down the myofibers. Supernatants were discarded, and pellets were suspended in 10 ml washing buffer (Ham's F10 medium containing 10% HS and 1% penicillin‐streptomycin) (pen/strep, Sigma‐Aldrich, St. Louis, MO). Pellets were triturated 10 times and allowed to incubate for 1 min to allow the clusters of nondigested fibers containing fibroblasts to fall to the bottom of the tube. Supernatants containing single fiber fragments were then transferred into a new 15 ml tube and centrifuged. After centrifugation, supernatants were discarded, 10 ml of washing buffer was added, and the pellet was triturated again 10 times and centrifuged. This step was repeated for a total of three washes. Fragmented myofibers were then digested in 3 ml of prewarmed Ham's F‐10 containing 10% HS, 0.5 U/mL dispase (Invitrogen), and 38 U/mL collagenase type II (US Biological, Salem, MA) in a 15 ml centrifuge tube for 30 min at 37°C with agitation. After digestion, 10 ml of wash buffer was added to the digest and satellite cells were liberated from the myofibers by trituration 10 times with a 20‐gauge syringe and centrifuged. Supernatants were filtered through 40‐µm sterile filters. The eluted flow‐through was centrifuged at 1,000 X g for 5 min to pellet satellite cells. Supernatants were discarded, and cells were suspended in 1 ml of Ham's F‐10 containing 20% fetal bovine serum (Genesee Scientific, San Diego, CA), 1% pen/strep, and 5 ng/ml basic fibroblast growth factor (Thermo Fisher Scientific, Gibco, Gaithersburg, MD). Cells were triturated 10 times to disperse and suspensions were quantified using a hemocytometer. Cells were seeded on collagen‐coated 12‐well plates at 0.1 × 10^6^ cells/well for proliferation assays, and on matrigel‐coated 6‐well plates at 0.1 × 10^6^ cells/well for differentiation studies. Plates were incubated at 5% CO_2_ at 37°C.

### 2.5 SC Proliferation Assay

#### 2.5.1 BrdU incorporation assay

Either 3 or 7 d after isolation, bromodeoxyuridine (BrdU) labeling reagent (Invitrogen, Carlsbad, CA) was added to each well at a 1:100 dilution. Cultures were incubated at 37°C for 1 hr, after which media were discarded and cell monolayers were washed once with ice‐cold PBS, fixed in 1 ml of ice‐cold 70% ethanol for 5 min at room temperature, and washed with PBS. After removal of PBS, plates were treated with 0.5 ml of 1.5M hydrochloric acid and allowed to sit at room temperature for 30 min. Plates were washed twice with PBS and blocked in PBS with 5% goat serum (Thermo Fisher Scientific) for 1 hr. Plates were then incubated with an anti‐BrdU antibody (clone G3G4, DSHB, Iowa City, IA), diluted 1:100 in PBS containing 5% goat serum. Plates were incubated overnight at 4°C. The following day, plates were washed three times with PBS, and a secondary antibody, Alexa Fluor 555 goat anti‐mouse IgG (Life Technologies, Eugene, OR) diluted 1:1,000 in PBS containing 5% goat serum, was applied. Cultures were incubated in the dark at room temperature for 2 hr. Plates were washed in PBS, and fluorescent mounting medium was added to each well. 4′,6‐diamidine‐2′‐phenylindole dihydrochloride (DAPI) counterstaining was used to identify nuclei. Images were collected using a Nikon ECLIPSE Ti‐E fluorescent microscope (Nikon Instruments Inc., Melville, NY). Number of nuclei positive for BrdU was quantified as a percent of total number of nuclei, and the percentage was used as an indicator for cell proliferation rate.

#### 2.5.2 Clonal Assay

To assess the proliferative capacity of SCs, we performed clonal assay. SCs isolated from NC and HFD muscles were cultured in growth medium in 10 cm dish for 3 d and 7d after isolation. SCs formed clones such that cells within each clone were derived from a single SC. Therefore, by counting the number of cells per clone, we obtained the information regarding the proliferative capacity of SCs in each treatment, i.e., the greater the number of cells per clone, the higher the proliferative capacity those cells possess.

### 2.6 Myogenic Differentiation

Cells plated for differentiation were cultured in growth medium for 7 days or until reaching a density of 95% confluence. Once cells reached confluence, the cells were washed 2X in PBS and released by putting 1 ml of 1:5 trypsin (Thermo Fisher Scientific) diluted in PBS in each well. Plates were incubated at 37°C until the cells detached from the bottom. Growth medium was added to neutralize the trypsin, and cells for each mouse were combined and centrifuged at 300 X g for 5 min to pellet cells. Supernatants were discarded, and 2 ml of growth medium was added to each tube. Cells were plated at confluence on matrigel‐coated 24‐well plates at 0.2 x 10^6^ cells/mL/well. To induce differentiation, growth medium was switched to differentiation medium (high‐glucose DMEM containing 3% horse serum and 1% pen/strep) the following day, and the cells were incubated at 37°C for 3 days. For immunocytochemistry, plates were washed once with ice‐cold PBS and fixed with 1 ml of ice‐cold 100% methanol at room temperature for 10 min, washed in PBS, blocked with 5% goat serum, and stained with antibodies against Pax7 (1:50 dilution, DSHB, Iowa City, IA), MyoD (1:200 dilution, Santa Cruz Biotechnology, Santa Cruz, CA), and/or myosin (clone MF20, DSHB, Iowa City, IA). After washing in PBS, the secondary antibodies (Alexa Fluor 555 goat anti‐mouse IgG and Alexa Fluor 488 goat anti‐rabbit IgG) and DAPI were applied to the cells at room temperature in the dark for 2 hr. After a brief wash in PBS, fluorescent mounting medium was added to each well. Images were taken using a Nikon ECLIPSE Ti‐E fluorescent microscope (Nikon Instruments Inc., Melville, NY).

### 2.7 Muscle oil red O staining

Cyrosectioned muscle samples on silane‐coated slides were allowed to dry at room temperature for 30 min prior to staining. Sections were placed in propylene glycol for 2 min and then incubated in concentrated Oil Red O solution for 6 min. Sections were then placed in 85% propylene glycol for 1 min. Slides were rinsed in distilled water and counter stained with hematoxylin for 2 min, rinsed with running water for 5 min, and then rinsed for 2 min in distilled water. Slides were mounted in Permount mounting medium (Thermo Fisher Scientific, Waltham, MA). Images were taken using a Nikon ECLIPSE 80i light microscope (Nikon Instruments Inc).

### 2.8 Muscle immunohistochemistry

Frozen muscle cyrosections were dried on silane‐coated slides for 30 min at room temperature prior to staining. Slides were washed once in PBS, then fixed in 4% paraformaldehyde for 10 min at room temperature. After three washes with PBS, muscle sections were permeabilized with 0.2% Triton X‐100 (Sigma‐Aldrich) in PBS at room temperature for 15 min, and washed two more times with PBS for 5 min each. Sections were incubated with wheat germ agglutinin (Thermo Fisher) and DAPI at room temperature in the dark for 1 hr. Sections were washed three times in PBS, then mounted with fluorescent mounting medium. Images were taken using a Nikon ECLIPSE Ti‐E fluorescent microscope (Nikon Instruments Inc). For image quantification, 10 fields was randomly chosen from each muscle section, and 100 fibers from each filed were analyzed.

### 2.9 Muscle single fiber isolation

*Gastrocnemius* muscles were isolated and digested in Dulbecco's modified Eagle's medium (Thermo Fisher Scientific) with high glucose, L‐glutamine, 110 mg/ml sodium pyruvate and 0.2% collagenase type I at 37°C for 1 hr. Digested muscles were then switched to DMEM containing 1% pen/strep and a large bore pipette was used to triturate the muscle and release myofibers from the muscle. A small‐bore pipette was used to transfer single fibers to wash media. Once a desired number of myofibers were collected, fibers were fixed in prewarmed 4% paraformaldehyde for 5 min, washed with PBS and permeabilized with 0.1% Triton X‐100 in PBS for 10 min. After incubation, two additional washes were performed with PBS and followed by an incubation in 5% goat serum in PBS for 1 hr. Fibers were incubated in Pax7 (DSHB, 1:50) and cleaved caspase 3 (Cell Signaling Technology, Danvers, MA) (1:200) antibodies, respectively, at 4°C overnight. The following day fibers were washed and incubated in secondary antibodies (Alexa Fluor 555 goat anti‐mouse IgG and Alexa Fluor 488 goat anti‐rabbit IgG) for 1 hr at room temperature in the dark. After three PBS washes, the sections were mounted with fluorescent mounting medium. Images were taken using a Nikon ECLIPSE Ti‐E fluorescent microscope (Nikon Instruments Inc).

### 2.10 Flow cytometry

*Gastrocnemius* muscles were minced and digested in collagenase B/dispase II for 1 hr with trituration every 15 min. Digestions were neutralized with FBS and pelleted at 350 X g. Samples used to analyze for SCs were stained with CD31‐APC, CD45‐APC, Sca1‐APC, and Vcam‐1‐biotin antibodies (BioLegend, San Diego, CA). After a brief wash, streptavidin‐PE‐Cy7 conjugated secondary antibody (BioLegend, San Diego, CA), together with PI (selection for dead cells) and calcein violet (selection for live cells) (BioLegend, San Diego, CA), was added prior to analysis. Samples were run through a FACSAria I (BD Biosciences, San Jose, CA), and data were analyzed using FlowJo software (FlowJo, LLC, Ashland, OR).

### 2.11 Muscle injury

The *tibialis anterior* and *gastrocnemius* muscles of mice were damaged by intramuscular injection of 50 µl and 300 µl cardiotoxin (CTX) dissolved in PBS to 0.1 mg/ml respectively. Prior to injection, mice were individually placed in an induction chamber and anesthetized using isoflurane. Isoflurane was administered at 3.5% in 100% oxygen with a flow of 1.0 L/min until loss of righting reflex. After induction, anesthesia was maintained through a facemask with 2% isoflurane in 100% oxygen at a flow rate of 1.0 L/min. Total loss of sensation was confirmed by pedal reflex (toe pinch) before CTX was injected into the animal's respective muscle. For multiple rounds of damage, the same protocol was followed with a month in between each round of damage and sample collection.

### 2.12 Statistical analysis

Data are presented as means ± standard error of the mean (S.E.M.), with significance set as * *p* < .05, ** *p* < .01, and *** *p* < .001. Data were analyzed using student's *t*‐test.

#### 2.12.1 Results

**Figure 1:**
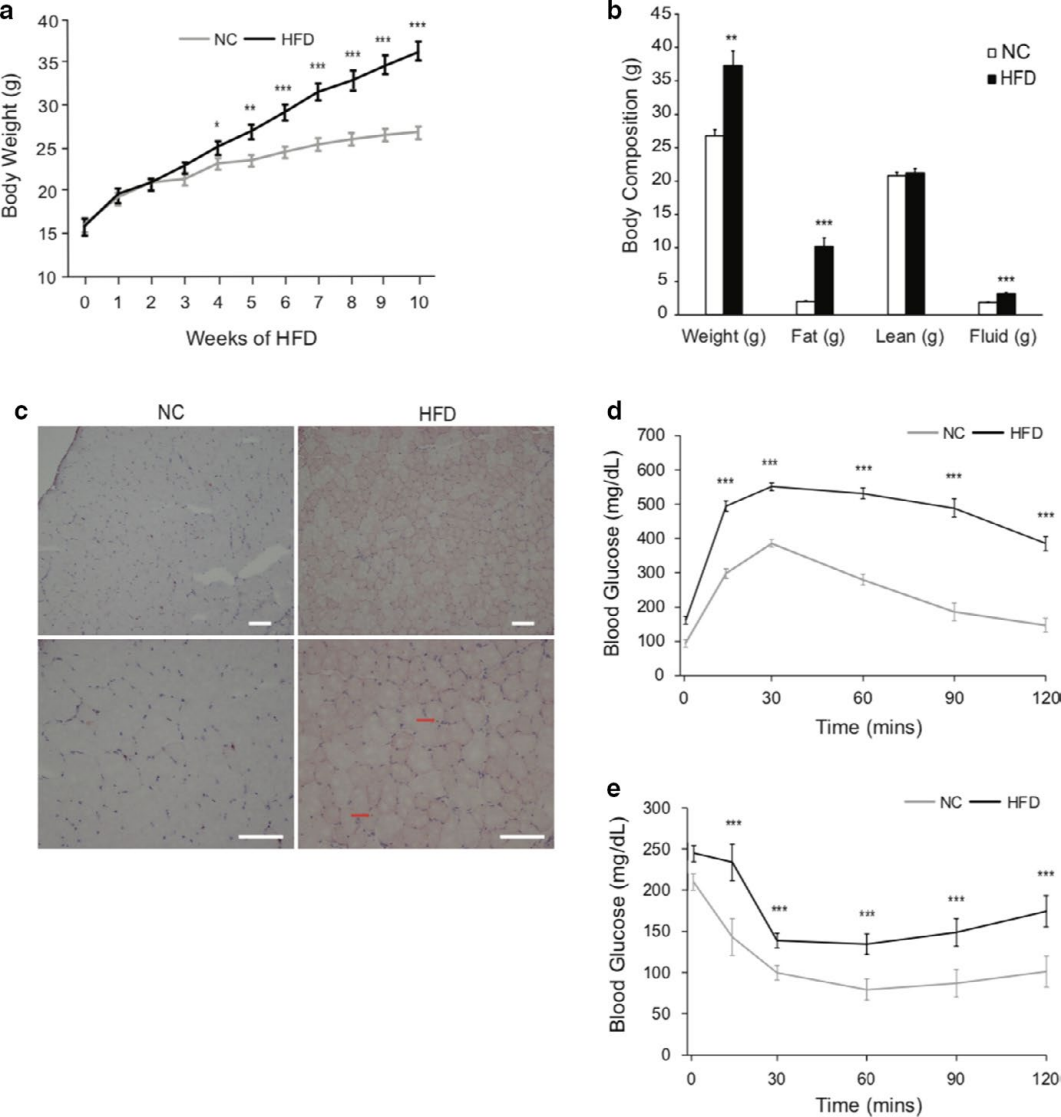
HFD mouse model shows increase in weight, fat accumulation, and insulin resistance. C57BL/6J mice were fed either NC or HFD at the age of 4–6 weeks for 10 weeks. (a) NC versus HFD mouse body weight over 10 weeks. (b) NC versus HFD mouse body composition after 10 weeks. (c) Oil Red O stain on TA muscle after HFD. Scale bars, 100 μm. Red arrows indicate intramyocellular lipid accumulation. *N* = 5 per treatment. (d) Glucose tolerance test (GTT). (e) Insulin tolerance test (ITT). *N* = 8 mice per treatment. Data represent means ± *SEM*. * *p* < .05, ** *p* < .01, *** *p* < .001 compared to NC

To evaluate SC function during an obese state, mice were subjected to an HFD feeding paradigm. Diets for the HFD mice consisted of 60% of total energy derived from fat. Mice were fed either NC or HFD starting at 4 weeks of age and were continuously fed this diet ad libitum for at least 10 weeks when an obese phenotype was observed. Mice fed HFD had greater (*p < *.001) body weights after 4 weeks of dietary intervention and this continued throughout the 10 week study (Figure 1a). Consistently, nuclear magnetic resonance (NMR) scans of the mice showed an increase in body fat mass in the HFD mice compared to the NC mice, but no differences were noted in skeletal muscle mass (Figure 1b). Oil Red O staining for lipid accumulation revealed an increase in lipid accumulation in the skeletal muscle of HFD mice in comparison to NC mice (Figure 1c). To test whether HFD induced insulin resistance, we performed glucose (GTT) (Figure 1d) and insulin tolerance tests (ITT) (Figure 1e) in mice fed HFD for 10 weeks. GTT and ITT tests clearly demonstrated that HFD‐fed mice had developed profound insulin resistance, compared to the control mice (Figure 1d and e). Together, these results validated the HFD mouse model for studying the impact of obesity on SC properties and function.

**Figure 2:**
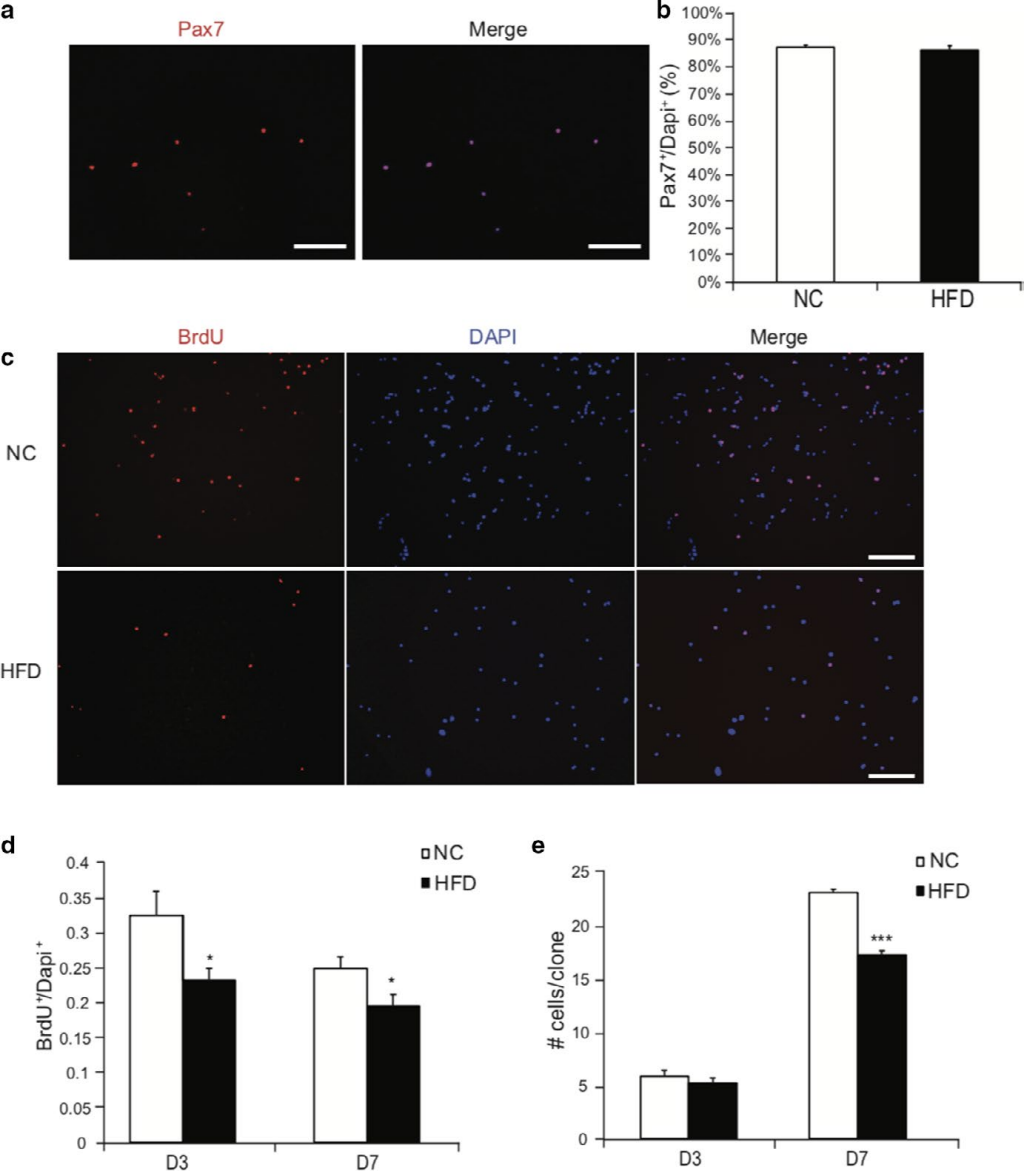
Satellite cells derived from HFD have impaired proliferative capacity *in vitro*. (a) Identification of SCs. 24 h after isolation, SCs were stained with Pax7 (red) and nuclei were counterstained with DAPI (blue). Scale bars, 50 μm. (b) Quantification of SCs as a percentage of total cells. (c) SCs were isolated from mice and cultured in growth medium for 3d or 7d. 10 μM of BrdU was added to the growth medium for 1h, cells were then fixed and stained with BrdU antibody. Nuclei was stained with DAPI (blue). Scale bars, 50 μm. (d) Quantification of BrdU^+^ cells expressed as percent of total number of nuclei. (e) Quantification of number of cells per clone 3 days or 7 days after isolation. *N* = 7 from each treatment. Data represent mean ± *SEM*. * *p* < .05, *** *p* < .001 compared to NC

To determine the effect of obesity on SC behaviors, we isolated and cultured SCs from muscles of mice exposed to different dietary treatments. Pax7 staining showed that over 90% of the isolated cells were SCs (Figure 2a and b). We then performed BrdU incorporation assay to evaluate SC proliferation in vitro. SCs derived from HFD‐fed mouse muscle had a reduced capacity to proliferate, compared to those from NC mouse muscle, as evidenced by reduced rate of BrdU incorporation in HFD SCs (Figure 2c and d). Clonal assay further confirmed this finding: although no difference in the number of cells per clone were evident at D3, by D7, fewer cells per clone (*p* < .001) were evident in SC cultures derived from HFD mice muscle (Figure 2e).

**Figure 3:**
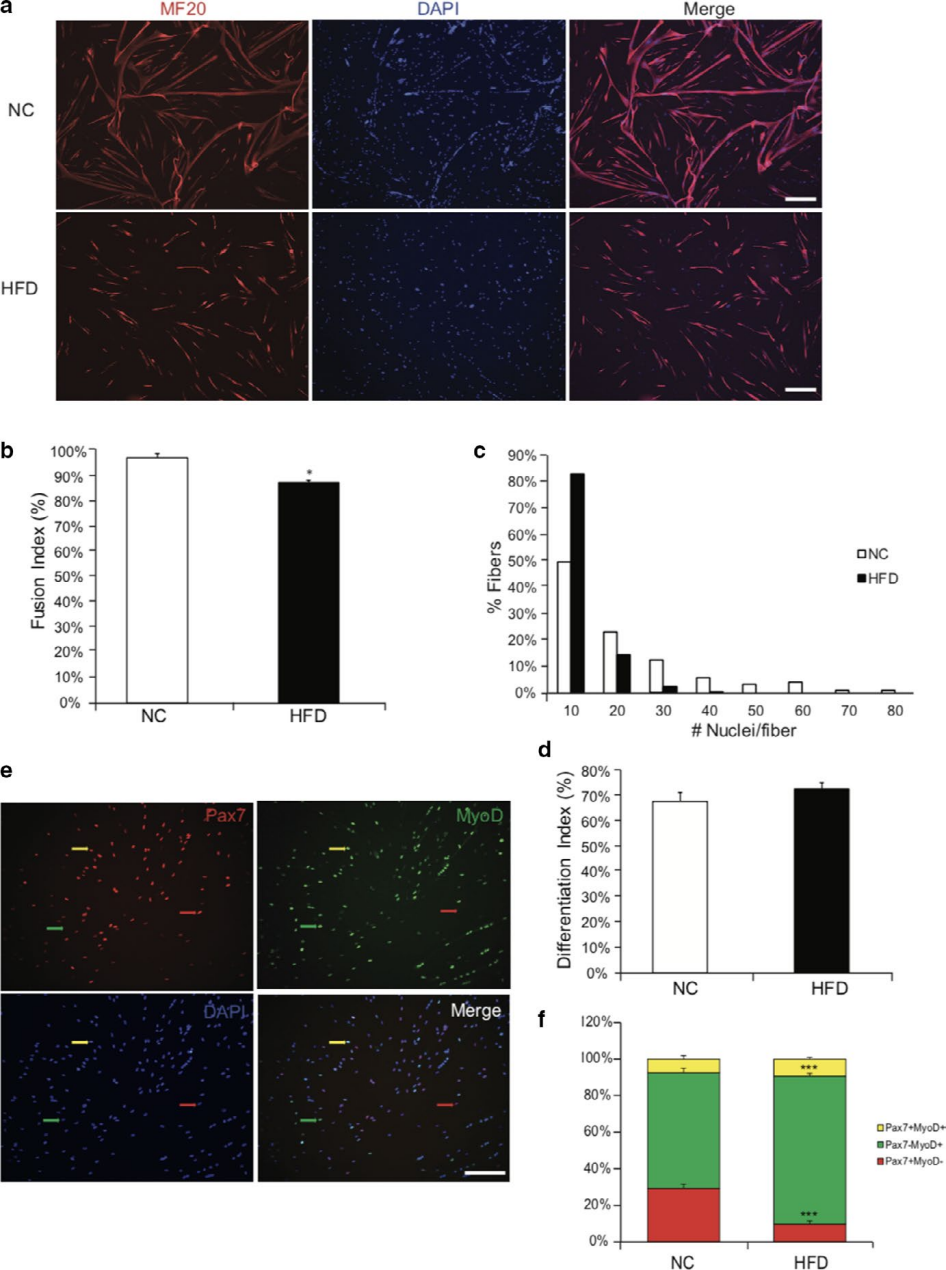
Satellite cells derived from HFD have impaired fusion capacities and self‐renewal. (a) SCs were isolated from NC and HFD mice and cultured for 7 days. Equal number of SCs were plated and induced to differentiate for 3 days. Myotubes were stained with myosin antibody (MF20, red) and counterstained with DAPI (blue) for nuclei. Scale bar, 200 μm. (b) Fusion index calculated as the ratio of the number of nuclei within myosin positive cells containing at least 3 nuclei to the number of nuclei within all myosin positive cells. (c) Histograms showing the distribution of the number of nuclei per fiber. (d) Differentiation index calculated as the ratio of the number of nuclei within myosin positive cells to the number of total cells. (e) Representative images showing the staining of Pax7 (red), MyoD (green), and nuclei (DAPI, blue). Red arrow, Pax7^+^ only; green arrow, MyoD^+^only; yellow arrow, Pax7^+^MyoD^+^. Scale bar, 50 μm. (f) SC subpopulation in NC and HFD after 3d differentiation. Pax7^+^, MyoD^+^, and Pax7^+^MyoD^+^ cell subpopulations were calculated as percent of total. *N* = 5 from each treatment. Data represent mean ± *SEM*. * *p < *.05, *** *p < *.001 compared to NC

To examine whether obesity impacted SC differentiation, SCs were isolated and allowed to reach confluence for 7 d. Once cells reached confluence, an equal number of SCs from NC and HFD were re‐plated and induced to differentiate for 3 d. After 3 d of differentiation, cells were stained with myosin heavy chain antibody (clone MF20) to identify myotubes (Figure 3a). SCs derived from muscle of HFD mice had reduced capacity to fuse into multinucleated (nuclear number ≥ 3) myotubes, compared to the NC group (Figure 3b and c). However, when the nuclei within all the myosin positive cells (including single‐ and bi‐nuclear cells) were counted as a percentage of total nuclei, there was no difference between HFD and NC treatments (Figure 3d). Because Pax7^‐^;MyoD^+^ cells represent committed myogenic SCs, and Pax7^+^;MyoD^‐^ cells represent SCs that have exited the lineage progression and have self‐renewed (Bentzinger et al., 2008; Gurevich et al., 2016), we next examined the expression of these myogenic markers on differentiated SCs. After 3 d of differentiation, SCs derived from HFD had fewer Pax7^+^;MyoD^‐^ cells (*p* < .001) and a corresponding increase in the percentage of Pax7^‐^;MyoD^+^ cells (*p* < .001), compared to SCs derived from NC mouse muscle (Figure 3e and f). Together, these findings suggest that SCs derived from HFD mice have impaired abilities to differentiate and self‐renewal, compared to those from NC mice.

**Figure 4:**
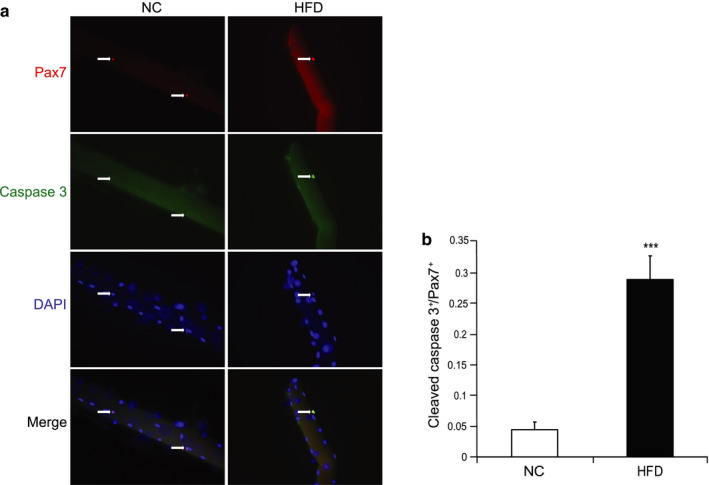
HFD induces SC apoptosis ex vivo. (a) Single fibers were isolated and fixed immediately. Representative images showing the staining of Pax7 (red), cleaved caspase 3 (green), and nuclei (DAPI, blue). (b) The ratio of the number of cleaved caspase 3^+^/Pax7^+^ cells over the total number of Pax7^+^ cells. *N* = 5 from each treatment. Data represent means ± *SEM*. *** *p* <.001 compared to NC

To determine whether HFD induces SC apoptosis, we isolated single muscle fibers from both NC and HFD mouse muscle and stained for cleaved caspase 3, a hallmark for cell apoptosis (Porter & Janicke, 1999). After isolation, single fibers were immediately fixed and stained with Pax7 and cleaved caspase 3 antibodies to identify SCs and their potential apoptotic subpopulation (Figure 4a). SC apoptosis was quantified as the ratio of Pax7^+^;cleaved caspase 3^+^ cells over the total number of Pax7^+^ cells per fiber. We found that SCs on HFD muscle fibers possessed a greater apoptotic subpopulation (*p* < 0.001) compared to those derived from NC mice (Figure 4b).

**Figure 5:**
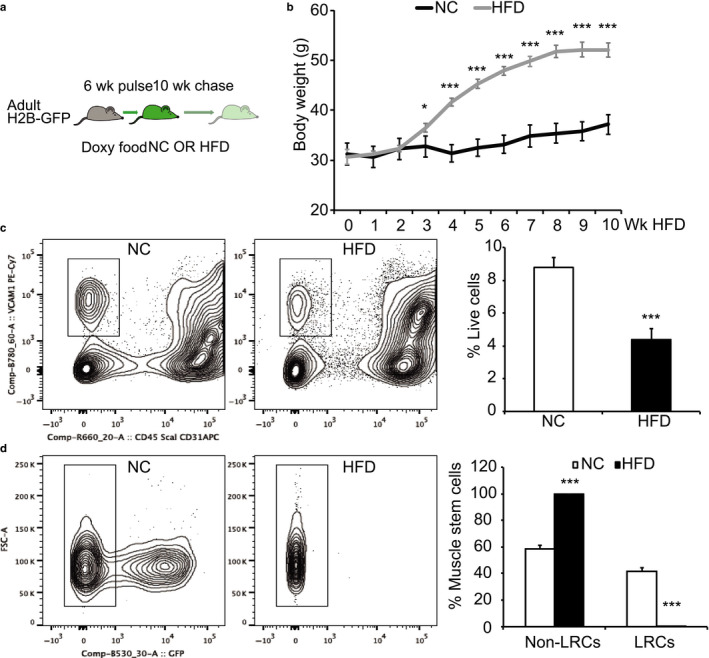
HFD reduces SC content and alters its properties in vivo. (a) Experimental scheme for in vivo lineage tracing. Adult mice, 7‐week‐old. NC, normal chow; HFD, high fat diet. (b) Body weight following HFD feeding. (c) FACS analysis of SC content based on surface markers VCam1^+^CD45^‐^CD31^‐^Sca1^‐^. (d) Non‐label retention cells (Non‐LRCs) and LRCs within SC population in NC and HFD mice. Data represent means ± *SEM* from *N* = 5 in (b), *N* = 4 in (c‐d) per treatment. *p < .05, ***p < .001 compared to NC treatment

To test whether the observed in vitro SC phenotype can be recapitulated in vivo, we studied SC properties in vivo using a H2B‐GFP mouse model. By administrating doxycycline chow, a green fluorescent protein (GFP)‐tagged histone 2B gene was expressed, and cells were labeled with green fluorescence, an engineered stable fluorescent dye that can only be diluted through cell division after doxycycline withdrawal (Kanda, Sullivan, & Wahl, 1998; Klochendler et al., 2012). After feeding mice with doxycycline for 6 weeks, we subjected these mice to either NC or HFD for 10 additional weeks and tracked the dilution of green florescence in SCs (Figure 5a). At the end of 10 weeks, body weights of HFD mice were significantly greater compared to those of NC mice (Figure 5b). We then isolated SCs and subjected them to fluorescence activated cell sorting (FACS) analysis and found SC number was greatly reduced in HFD muscle (*p* < .001) (Figure 5c). We then measured the GFP intensity of SCs derived from NC and HFD muscles and found that although NC mice appeared to retain a greater population of GFP^+^ SCs, HFD SCs lost almost all of the GFP signal (Figure 5d). Together, these data show that HFD reduces SC content and increases its cycling properties in vivo.

**Figure 6:**
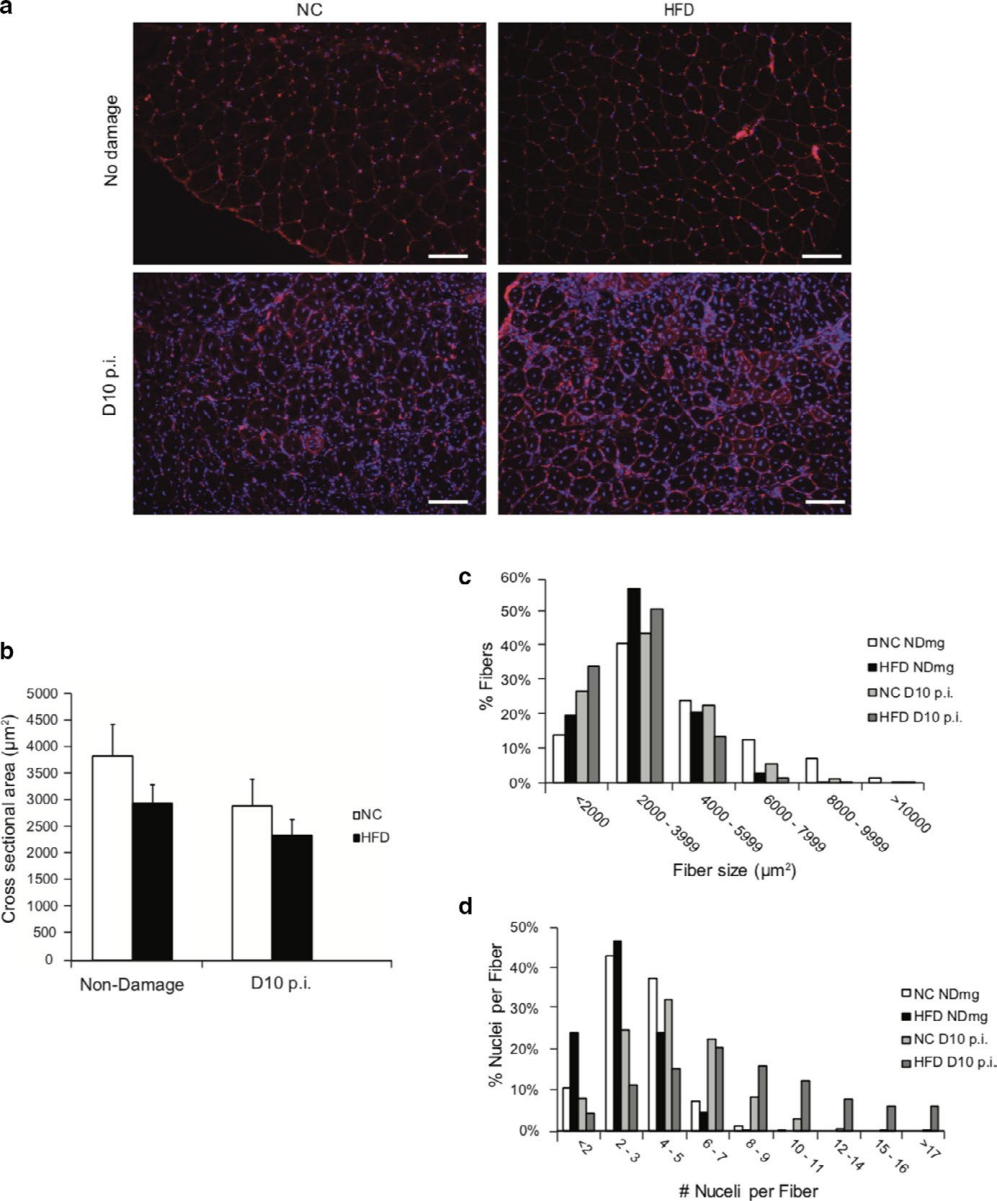
HFD mice exhibit slight impairment in muscle regeneration. TA muscles from mice were injected with 50 μl caridotoxin to induce injury. Muscle samples were harvested 10 d post injury. (a) Muscle fiber membrane was stained red with wheat germ agglutinin (WGA), nuclei were stained blue with DAPI. Scale bars, 100 μm. (b) Muscle fiber cross‐sectional area (CSA) of the fibers containing central nuclei (newborn fibers). (c‐d) Frequency distribution of the CSA values (c) and the number of nuclei per fiber (d) in the newborn fibers. *N* = 4 from each treatment. Data represent mean ± *SEM*

**Figure 7:**
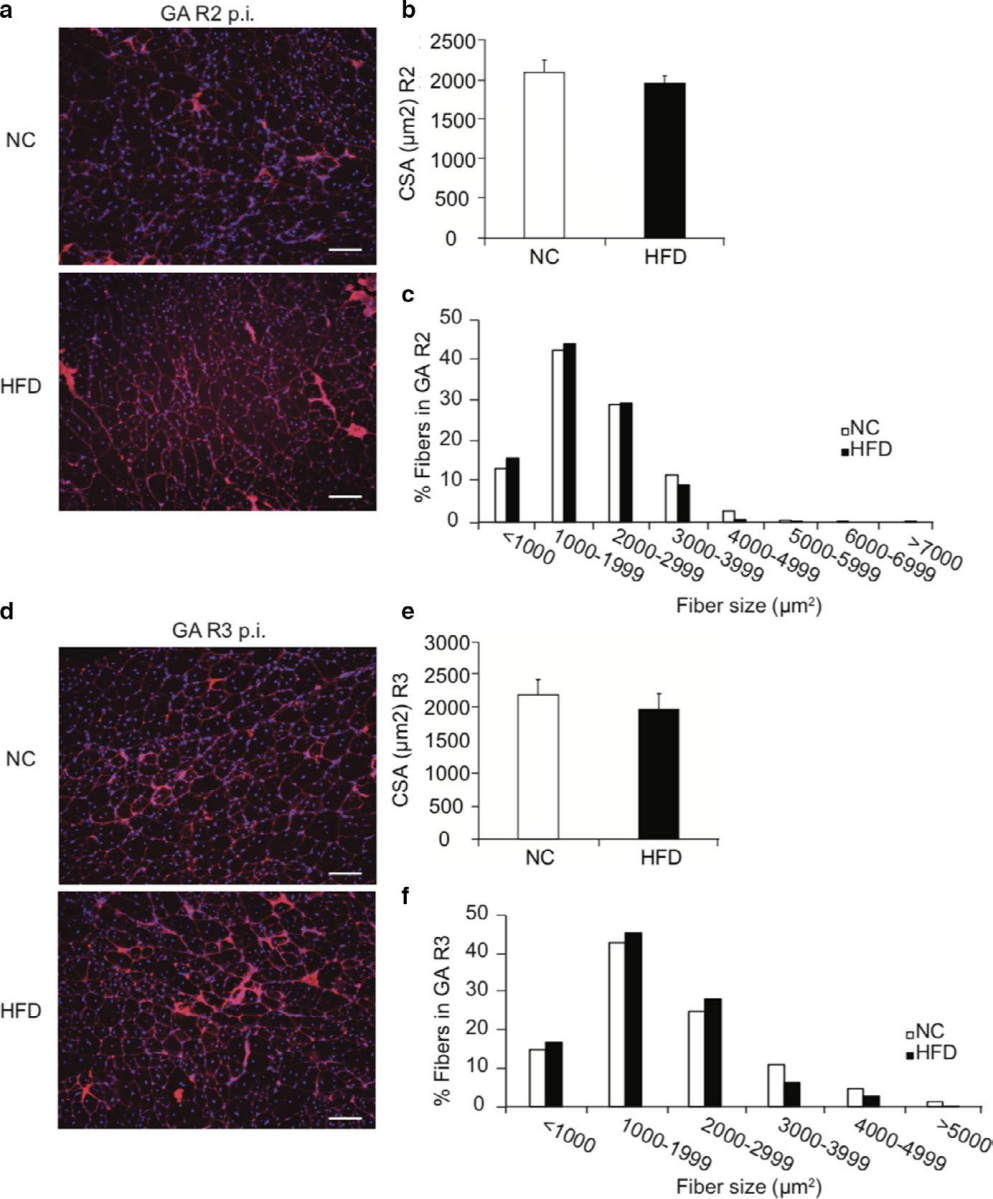
Muscle regeneration is not affected by HFD in multiple rounds of damage. Left GA muscles had three rounds of damage (R3) and right GA muscles had two round of damage (R2) with 4 weeks in between each round of damage. (a, d) WGA staining of muscle fiber membrane and DAPI staining of nuclei in 2nd‐ and 3rd‐round regenerated muscle fibers. Scale bars, 100 μm. (b, e) Muscle fiber cross sectional areas (CSA) of the regenerated muscle fibers. (c, f) Frequency distribution of the CSA of the regenerated fibers. *N* = 5 per treatment

To assess whether the negative impacts of obesity on SC properties led to impaired muscle regeneration, we used cardiotoxin (CTX) to induce muscle damage in both NC and HFD TA muscles. Ten days after damage, TA muscles were collected and stained (Figure 6a) to quantify muscle fiber cross sectional area (CSA) of the regenerating fibers containing centrally localized nuclei (Figure 6b). Although we observed a trend of decrease in the CSA 10 d post injury (Figure 6b) in regenerating HFD muscle fibers compared to NC fibers, the difference was not statistically significant (*p* > 05). Frequency histograms of muscle fiber CSA showed that HFD muscles contained a higher subpopulation of fibers with smaller CSA, compared to NC muscles (Figure 6c). Interestingly, HFD muscles appeared to contain fibers with more nuclei (Figure 6d). Together, these findings suggest that the negative impact of obesity on muscle regeneration was mild. To further test whether subtle effects of obesity on muscle regeneration could be exacerbated by prolonged muscle injury and recovery, we subjected the NC and HFD mice to a second and third round of muscle injury. Even more surprisingly, we found that there was no differences in the CSA of the regenerated fibers in either the 2nd (Figure 7a, b, and c) and 3rd (Figure 7d, e, and f) round of muscle regeneration.

## 3 DISCUSSION

Obesity without doubt causes major shifts and alterations in body composition and metabolism (Schiaffino, Dyar, Ciciliot, Blaauw, & Sandri, 2013). Mice fed a high fat diet show considerable changes in fat mass, body weight, and a noticeable lipid accumulation in muscle fibers. Moreover, obesity results in decreases in skeletal muscle metabolic flexibility, as well as a decreased ability to regenerate after damage (Goodpaster, Thaete, & Kelley, 2000; Hu et al., 2010). Our work contributes to and extends the literature by providing evidence that HFD impairs SC proliferation, differentiation, and self‐renewal in vitro as well as compromises SC properties in vivo. However, we failed to show that such effects impact muscle regeneration in vivo.

After 3d of culture, SCs derived from HFD mice have a notable decrease in BrdU^+^ cells, indicative of dividing cells, although no differences in cell number are observed at this time. While it takes SCs nearly 48 hr to activate and begin proliferating after isolation (Kuang & Rudnicki, 2008), it is possible that we have captured a time point during which SCs are progressing through the latter stages of the cell cycle but not yet undergone mitosis. This time point could highlight a change in the rate through the cell cycle, but not yet depict changes in SC number. Furthermore, SCs derived from mice fed HFD have diminished capacities to fuse as evidenced by decreases in the fusion index and the number of nuclei per fiber, even though the differentiation indices, defined as the ratio of the number of nuclei within myosin positive myotubes to the total nuclear number, are comparable between NC and HFD SCs. This impairment in SC fusion may be a result of decreased expression of MyoD, myogenin, and myosin heavy chain, as reported previously (Tamilarasan et al., 2012), or could be linked to chronic inflammation associated with obesity (Arnold et al., 2007; Saclier et al., 2013). Regardless, these results bring to light that SC properties are altered by high fat diets, as NC and HFD muscles are cultured in the same conditions. Although the exact mechanism of the compromised capacities for SCs to proliferate, fuse, and self‐renew merits further investigation, these data suggest that SCs possess some sort of “obese” imprint established by their original microenvironment or niche.

These negative impacts observed in vitro are corroborated by in vivo and ex vivo data

First, our FACS data show that dietary obesity decreases SC content in vivo. This decrease may be caused by obesity‐induced apoptosis, primarily because HFD fibers contained a proportion of SCs that were positive for cleaved caspase 3. This result may help explain why SCs in vitro have impaired proliferation, as indicated by clonal and BrdU incorporation assays. Although it is unclear what causes an increase in SC apoptosis on muscle fibers, various factors associated with obesity could be involved. For example, with obesity there is a noted elevation in reactive oxygen species (ROS), oxidative stress, and mitochondrial dysfunction, which could play a role in increased apoptotic SCs in HFD mice (Bonnard et al., 2008). Second, results from our in vivo GFP tracking experiment indicate that the enhanced SC cycling rate, as indicated by a more rapid loss of GFP, may reflect a lack of quiescent status of SCs in the metabolically unfavorable obese niche. It is reasonable to postulate that since quiescence is a hallmark of adult stem cells in general, lack of “sleeping” status could exert great cellular stress on SC. Such a negative impact may trigger SC apoptosis or, in parallel with other factors‐induced apoptosis, reduce SC pools. Yet, the length of time SCs tolerate or resist modification from an obesity‐induced stress remains an enigma. Although the exact mechanism for the effects of HFD on SCs remains elusive at this stage, it is clear that HFD creates an adverse niche for SCs to survive. For example, there is an increase in M1 macrophages associated with obesity, which may be responsible for the secretion of pro‐inflammatory cytokines such as TNF and IL‐6 (Domingues‐Faria, Vasson, Goncalves‐Mendes, Boirie, & Walrand, 2016; Zhang et al., 2013). Thus, chronic inflammation caused by macrophages and other infiltrating immune cells may provide an adverse microenvironment for SCs to survive.

Based on the literature, we believed that obese patients faced with a muscle injury may experience some level of delayed recovery. Yet, evidence provided from this study suggest otherwise. Results of our chemical‐induced muscle regeneration show that although there may be some minor effects on muscle recovery in obese patients, impacts are quite mild. Although our experiments only consisted of three‐rounds of injury and regeneration, these data support the argument that insults exerted by HFD on SCs may still reside within the range of SC capacity to handle muscle regeneration, at least in the short‐term. This hypothesis is supported by findings from other laboratories. For example, long‐term high fat feeding (8 months) results in a marked decrease in TA muscle regeneration, as demonstrated by a reduction in muscle mass, smaller myofibers, increased collagen deposition, and larger interstitial spaces in comparison to NC mice (Hu et al., 2010). Another study using a shorter high caloric feeding paradigm (3 weeks) showed similar results in young mice aged 3–6 weeks old (Woo et al., 2011). However, these findings are not repeatable in HFD models fed for an intermediate amount of time. For example, a study feeding high fat diets to mice for 12 weeks, a time frame similar to that reported herein, failed to observe a marked decrease in the size of regenerating fibers after inducing injury of the *extensor digitorum longus* (EDL) with cardiotoxin (Nguyen, Cheng, & Koh, 2011). These findings are consistent with our data, showing only a mild impairment in muscle regeneration after an intermediate feeding time period. The only difference in the two studies was we studied the TA rather than the EDL. While both the TA and EDL consist of primarily fast‐twitch IIB fiber types, the TA consists of a larger proportion of IIA fibers and thus could have an impact on muscle regeneration after injury (Bloemberg & Quadrilatero, 2012). Regardless of the muscle type differences, our results and that from Nguyen, et al. indicate that a mild impairment in HFD‐feeding suggest a compensatory mechanism may exist to help regenerate muscle after an insult. Because muscle regeneration is a complex process which involves a hierarchy of cellular events, including but not limited to SCs, it is reasonable to speculate that the negative impacts of HFD on SCs may be somehow buffered in vivo as compared to in vitro. For example, HFD mice have insulin resistance and exhibit hyperinsulinemia with a high level of circulating insulin, a well‐known myoblast proliferation and differentiation enhancer (Allen & Rankin, 1990; Brown et al., 2016; Prentki & Nolan, 2006), which may boost SC function and thus compensate HFD‐caused SC harm in vivo, at least in a temporary manner. Thus, future studies focusing on the signaling pathways emanating from the HFD niche will help expound the molecular mechanism responsible for SC homeostasis in a given pathological setting, which, in our case, is obesity.

## 4 SUMMARY

In summary, this study reveals that obese niche has a negative impact on SCs in vitro and in vivo*;* however, the seemingly negative impacts on SCs has mild effects on adult regenerative myogenesis in vivo, even after multiple rounds of muscle injury. These results suggest that in vivo mechanisms may exist, perhaps at the whole body level, that can mitigate or overcome damage to muscle stem cells. Further defining these mechanisms may lead to strategies to ameliorate the potential negative impacts of obesity on adult stem cells, and thus improve the quality of life and physical well‐being in the obese patients.

## 5 ACKNOWLEGMENTS

The authors thank undergraduate student researchers, Nan Yang and Kelly M. Ramos for their efforts in helping collect data.

## CONFLICT OF INTEREST

The authors declare no potential conflict of interest.

## AUTHOR CONTRIBUTIONS

A.E.G., H.S., and D.E.G. designed the experiments and prepared the manuscript. A.E.G., M.R.D., C.Y., K.M.R., N.Y., and L.K. performed the experiments and analyzed the data. All authors contributed to the manuscript discussion and revision.
